# Evaluation of Gentamicin Release of PMMA Cements Using Different Methods: HPLC, Elution and Inhibition Zone Testing

**DOI:** 10.3390/antibiotics13080754

**Published:** 2024-08-11

**Authors:** Clemens Kittinger, Johannes Stadler, Klaus Dieter Kühn

**Affiliations:** 1 D&R Insitute of Hygiene, Microbiology and Environmental Medicine, Medical University of Graz, Neue Stiftingtalstraße 6/III 1, 8036 Graz, Austria; clemens.kittinger@medunigraz.at; 2 Department of Orthopaedics and Trauma, Medical University of Graz, 8036 Graz, Austria; johannes.stadler@ooeg.at

**Keywords:** PMMA cement, antibiotic elution, gentamicin, eluates and discs, area zone, HPLC

## Abstract

(1) Background: There is an ongoing discussion on the elution efficacy of antibiotic-impregnated cements. Our experiments were intended to clarify if there are differences in the antibiotic elution of HPLC compared with inhibition zone testing using eluates or PMMA discs. (2) Materials and Methods: Two cement brands with different concentrations of the active ingredient were tested in antimicrobial Kirby–Bauer (disc diffusion) assays. Cement platelets were directly applied on the agar plates and their zone of inhibition was measured. In parallel, the platelets were incubated in phosphate buffered saline (PBS) and at distinct points of time transferred into new buffer. At these time points, 50 µL of the bone cement eluates was used for zone of inhibition testing. Standard gentamicin sulfate solutions served as a control in the same test setup. To verify the microbiological investigations, the antibiotic content of the eluates was also measured via high-performance liquid chromatography (HPLC). (3) Results: The experiments with cement eluates showed better differentiable results than the direct application of the cement discs. The results were also comparable to investigations with HPLC and gentamicin sulfate standard solutions. (4) Conclusions: The results of elution rates are influenced by the test system and the period of observation chosen. The microbial test systems reflect the results of HPLC to the same degree and give evidence of the efficacy of the antibiotics. The HPLC tests on eluates were more suitable in representing differences in release characteristics.

## 1. Introduction

Polymethymethacrylate (PMMA) cements with antibiotics are very important in orthopedic surgery to prevent early stages of infection or the colonization of implants and for prophylaxis or adjuvant therapy of infected revisions [1]. Continuous local application of antibiotics improves the outcome of periprosthetic joint infections [2] because of high local concentrations [3,4,5,6]. Tissue irritation and immune dysfunction facilitate bacterial invasion and the colonization of implant surfaces immediately after surgery. Polymicrobial infections and changes in the bacterial spectrum as well as growing resistance have to be considered for treatment options and might favor high local antibiotic concentrations [1,7]. Therefore, antibiotic-containing cements should have the highest possible release rates at the beginning followed by an elution over a period of some weeks at spacer application [8,9,10,11] to help the body fight bacteria [12]. In clinical practice, aminoglycosides, especially gentamicin sulfate (GS), is the most commonly used antibiotic in PMMA cements [1,13].

Adding antibiotics to the cements alters the mechanical properties of the cements [10]. From a chemical point of view, the antibiotic particles are not included in the polymer chains and act as foreign matter within the cement matrix [13]. On the one hand, this is important for the release characteristics; on the other hand, too-high amounts of antibiotics can significantly weaken the strength of the matrix. [14,15,16]. However, the amount of antibiotics in commercially produced cements is between 1.25 and 2.5% *w*/*w* in the polymer powder and does not affect their stability [17,18]. The added antibiotics are only partially released (approx. 10%), but a high local antibiotic concentration should still be built up [19]. On long-term observation, the antibiotic-containing cements release steadily in subinhibitory concentrations [20,21,22].

The release of the active ingredients from the PMMA matrix is a diffusion process, directly related to the size of the cement surface [19,23]. Another factor affecting the release of the antibiotic is the absorption of water or body fluids by the cement. The liquid uptake depends on the hydrophilic properties, the roughness and the porosity of the material. The liquids can get into the pores and wash off the surface-incorporated antibiotic [9,24,25,26]. In addition, the release behavior depends on properties of the cement [24,27,28].

Many studies indicate different release properties of antibiotic-containing cements depending on preparation or brand [19,29]. We tested the release properties of two commercially available bone cements: one contains 1 g (2.5%) whereas the other contains 0.5 g (1.25%) gentamicin sulfate (The Norwegian Arthroplasty Register, Table 1). We applied different test methods but the same specimens and sample preparation to take methodical influence into account.

## 2. Results

### 2.1. Testing PMMA Discs on Agar Plates with S. aureus

**Figure 1:**
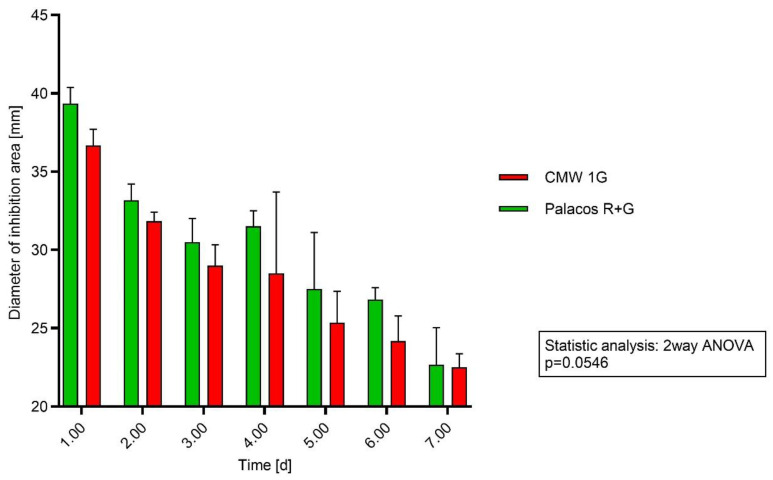
Testing method agar diffusion: Cement discs were applied directly on agar plates streaked with *S. aureus* agar. Every 24 h, the discs were transferred onto new agar plates. The diameter of inhibition area is in millimeters.

The diameters (y-axis [mm]) of the inhibition areas of Palacos R+G and CWW 1G over 7 days (x-axis) using *S. aureus* showed in each case a larger area for Palacos R+G. The diameters of the inhibition areas of Palacos R+G and CWW 1G did not show significant differences (*p* = 0.0546) over the 7-day test period. The results did not reflect the amount of antibiotics within the PMMA cements (Figure 1). 

### 2.2. Testing PMMA Discs on Agar Plates with S. epidermidis

Diameters (x-axis [mm]) of the inhibition areas of Palacos R+G and CWW 1G over 7 days (y-axis) using *S. epidermidis* showed in each case a larger area for Palacos R+G. The diameters of the inhibition areas of Palacos R+G and CWW 1G did not show significant differences (*p* = 0.0372) over the 7-day test period. The amount of the antibiotic was also not reflected by the inhibition areas.

**Figure 2:**
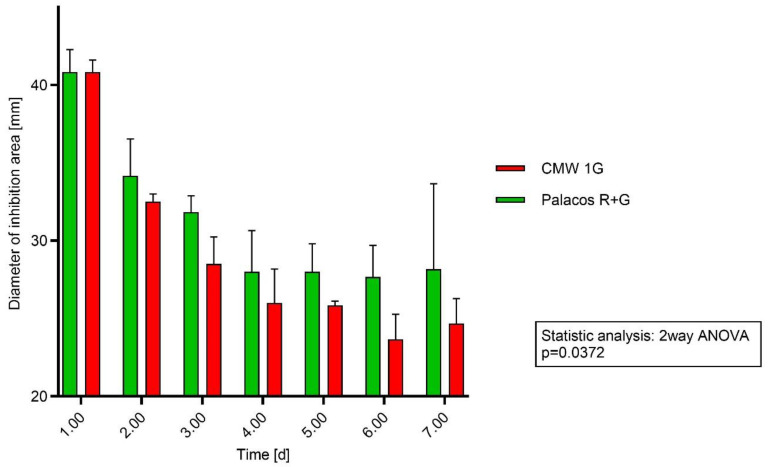
Testing method agar diffusion: Cement discs were applied directly on agar plates streaked with *S. epidermidis* agar. Every 24 h, the discs were transferred onto new agar plates. The diameter of inhibition area is in millimeters.

The released gentamicin out from the bone cement matrix was more effective against *S. epidermidis* compared to *S. aureus* (Figure 2).

### 2.3. Agar Diffusion Test with Gentamicin Sulfate Solution

Two acrylic cements with completely different compositions that showed differences in their release in HPLC measurements and contained different concentrations of GS (0.5 g and 1.0 g) were chosen for evaluating this test setup. To measure diffusion, 50 µL of eluates of cement discs of CMW 1G and Palacos R+G was directly applied to holes in the middle of agar plates and then incubated for 24 h at standard conditions.

**Figure 3:**
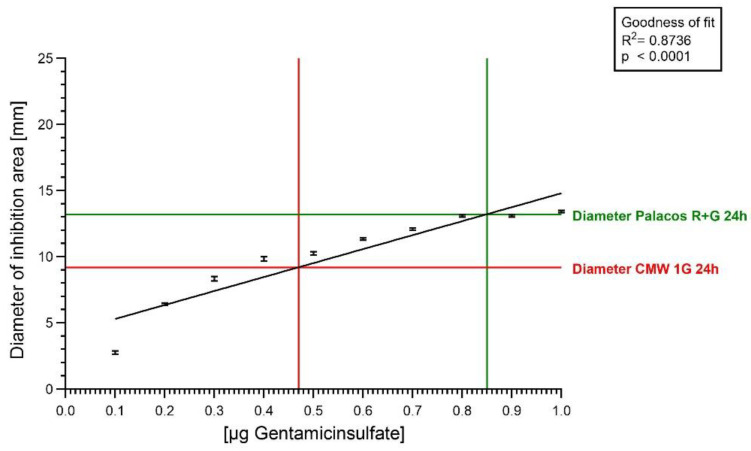
Linear regression line. The black line is the regression line. Diameter of inhibition zone in mm for Palacos R+G (green) and CMW 1G (red) in 24 h.

The results of agar diffusion testing in inhibiting areas of eluates of gentamicin containing cement discs reflected differences in the cements, whereas the direct application of bone cement discs on agar did not. The diameters of the 24 h values of the figure above were applied in the standard curve (Figure 3) to determine the released GS amounts applied for linear regression.

**Figure 4:**
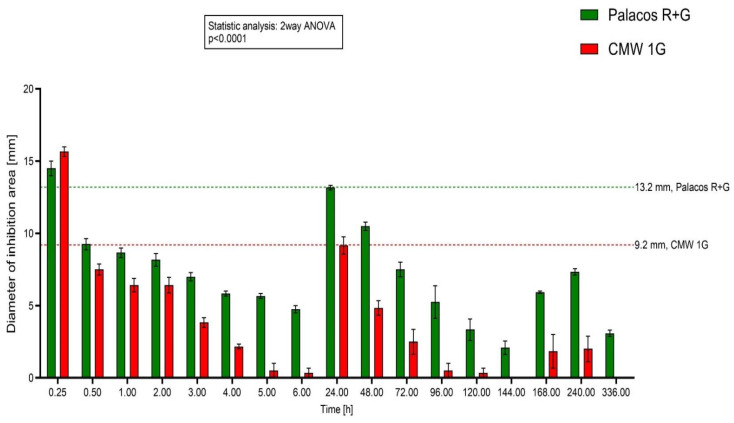
Testing eluates: Diameter of Inhibition Areas of Palacos R+G (0.5 g) and CMW 1G (1.0 g) cements over 14 days, y-axis shows the diameters of inhibition areas [mm], x-axis represents the time scale [hours]. Values of the measured diameters differ significantly at the desired time points.

Data were fitted to the diameter of the applied GS standard solutions. The y-axis shows the diameter of the area of inhibition [mm], and the x-axis represents the GS concentration [µg]. The measured diameters of the inhibition areas were applied for linear regression and resulted in a very good fit (R^2^ = 0.8736). For diameters in the range of 6–15 mm, the amount of released GS could be calculated. The green and the red line starting on the y-axis indicate the zone of inhibition area diameter and their corresponding GS concentration on the x-axis for the 24 h value in Figure 4.

### 2.4. Analysis of Bone Cement Eluates with HPLC

All tested antibiotic-containing specimens showed a similar pattern of release. Within the first hour, they showed high antibiotic release followed by a prolonged elution on a lower level over time. Palacos R+G with 1.25% GS showed higher values of cumulative released GS, whereas CMW 1 G with 2.5% GS showed less released antibiotics over time in the HPLC tests with eluates.

**Figure 5:**
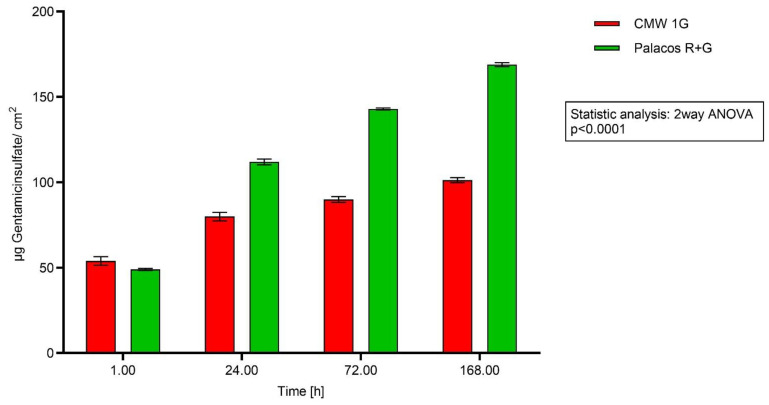
Testing elution with eluates: Accumulative elution of GS out of cement discs.

Three independent samples were measured, and release was calculated per cm^2^. The y-axis shows the amount of GS [GS/cm^2^], and the x-axis represents the time scale [h]. There were significant differences at the different time points in the release characteristics of the PMMA cements tested (*p* Value < 0.0001) (Analytic Center Berlin, AZB) (Figure 5).

## 3. Materials and Methods

### 3.1. PMMA Bone Cements

The bone cements tested were CMW 1G (DePuy/JJ, MMA homopolymer, 1 g gentamicin in 40 g powder) and Palacos^®^ R+G (Heraeus Medical, MMA/MA copolymer, 0.5 g gentamicin in 40 g powder).

### 3.2. PMMA Cement Discs

Small discs with a diameter of 15 mm and a thickness of 3 mm were created according to the manufacturers’ instructions. All cements were mixed under atmospheric pressure and room temperature (RT, 23 °C, humidity 34%) for the given time in the manufacturers’ manuals. The doughy cement was then immediately transferred into silicon molds and covered with a plastic film. The silicon molds were then covered with a constant weight. After 20 min, the hardened cement discs were taken out of the silicon molds and stored in small plastic containers at room temperature for at least 48 h until use.

They were additionally selected according to the following characteristics:

Macroscopic even surface.

Exact diameter of 15 mm.

Weight between 0.560 and 0.575 g (analytic balance, Cubis, Sartorius Austria).

### 3.3. Bacterial Strains

For the agar diffusion tests in this study the following bacteria were used:

*Staphylococcus aureus* DSM 799 (*S. aureus*).

*Staphylococcus epidermidis* DSM 1798 (*S. epidermidis*).

The strains were tested for their susceptibility to gentamicin with an E-test (Biomerieux, Austria). The E-test was carried out three times and showed a sensitivity of 0.25 µg/mL for GS for *S. aureus* and 0.125 for *S. epidermidis*. All experiments were conducted on Mueller–Hinton plates with a standard agar volume (according to actual EUCAST criteria, 2024). Agar plates were always incubated at 36 °C ± 1 °C for 24 h.

### 3.4. Preparation of Bacterial Suspension

Agar plates were plated with a bacterial suspension with a density of McFarland 0.5 turbidity standard (around 1.5 × 10^8^ cfu/mL).

### 3.5. Agar Diffusion

For the first test, PMMA cement discs were used directly. Bacteria were swabbed over Mueller–Hinton agar plates. The cement discs were placed in the center of the agar plates and incubated at 37° Celsius for 24 h. The same cement discs were placed on new agar plates and incubated again for 24 h. This procedure was carried out for all cements for 6 consecutive days. Of every cement, three discs were tested in parallel (Table 1).

### 3.6. Elution Tests

Discs were eluted in 10 mL phosphate buffered saline (PBS, Biochrom, Vienna, Austria). The discs were dropped in, swayed once and stored at RT. The discs were transferred at the desired time points with forceps into fresh buffer. To avoid displacement of the antibiotic to the next tube, the discs were briefly dabbed on both sides. Discs were transferred after 0.25, 0.5, 1, 2, 3, 4, 5, 6 and 24 h and further on after 2, 3, 4, 5, 6, 7, 10 and 14 days (Table 1).

Agar plates were prepared like above, and after plating the bacteria suspension, a 6 mm diameter hole was pricked into the middle of the plate. Then, 50 µL of the eluates was transferred into the hole for the agar diffusion test.

### 3.7. Agar Diffusion Test with Gentamicin Sulfate Solution

A gentamicin sulfate (Sigma Aldrich, Austria) standard solution ([1 mg/mL]) was diluted to obtain final concentrations from 0.1 to 1.0 µg/50 µL in 0.1 steps. Agar plates were prepared like above, and after plating the bacteria suspension, a 6 mm diameter hole was pricked into the middle of the plate. Then, 50 µL of the standard solutions was transferred into the hole (Table 1).

### 3.8. Measurement of the Inhibition Areas

In all experimental settings, the areas of inhibition were always measured directly after incubation. The diameters were measured twice (2nd measurement vertical to the 1st), and the mean was then calculated.

### 3.9. HPLC (Carried Out at Analytic Center Berlin, AZB)

To ascertain the amount of released antibiotic over time, eluates of the cement discs (produced as described above) were analyzed using HPLC. The discs were eluted at RT in 10 mL dissolution medium (PBS). Aliquots were taken, and the dissolution medium was renewed at the following sampling points: 1 h, 24 h, 72 h and 164 h. The dissolution medium samples were stored at −20 °C until analysis.

Ten calibration standards from 100 to 7500 ng/mL (for gentamicin) as well as a blank sample (blank with internal standard) were prepared by spiking 200 µL of the working solutions with internal standard working solutions (18 µL gentamicin).

The study samples were diluted by a factor of 20 and prepared according to the calibration standards by adding an internal standard working solution. Every sample was analyzed three times (Table 1).

LC-MS/MS chromatographic separation was performed on a modular HPLC 1200 Series (Agilent Technologies, Waldbronn, Germany) using a Luna C18 (II) column, 150 × 2 mm, with two C18 4 × 2 mm guard columns (Phenomenex, Aschaffenburg, Germany) at 25 °C. The injection volume was 2 µL. The mobile phase was 0.11 M trifluoroacetic acid/methanol (50:50), and mobile phase B was acetonitrile. An isocratic separation for gentamicin was achieved with an A:B ratio of 95:5 at a flow rate of 0.25 mL/min. The run time was 2.5 min, and the total cycle time was less than 3 min. Under these conditions, the four gentamicin components C1, C2, C2a and C1a co-eluted. The HPLC method was previously used by Heller et al. (2005) to detect gentamicin in biopsy samples [30]. The detection of the co-eluted gentamicin components was carried out using an API 4000 QTrap (Applied Biosystems, Darmstadt, Germany). Ionization was carried out with an electrospray interface (positive polarity) using a mass selective detector (Applied Biosystems Darmstadt Germany) in multiple reaction monitoring mode (MRM). The extracted ion chromatograms of the following ion transitions were stored and calculated: 478.4 → 322.3 *m*/*z* (gentamicin C1), 464.4 → 322.3 *m*/*z* (gentamicin C2 and C2a), 450.3 → 322.3 *m*/*z* (gentamicin C1a.) and 468.4 → 163.1 *m*/*z* (internal standard). The three ion transitions of the gentamicin components were summed by the software Analyst 1.4.2 (Applied Biosystems, Darmstadt, Germany) and calculated with Excel (Microsoft, Unterschleißheim, Germany).

### 3.10. Data Analysis

For all data, the mean and standard deviation of the mean were calculated, and data were analyzed with GraphPadPrism™ 5.01 for Windows. Graphs were calculated with GraphPadPrism™ 10.0 for Windows, GraphPad Software, San Diego, CA USA, www.graphpad.com (accessed on 1 July 2024).

## 4. Discussion

Directly applying cement discs to agar plates and measurement of the area of inhibition was chosen as a method to demonstrate a direct connection to the microbiological efficacy of the added antibiotic under reproducible settings [31,32]. In a study on 5 different PMMA cements carried out by Squire et al. (2008) and Meyer et al. (2011) [33,34], differences in their inhibition zones were mentioned (when using plate diffusion), as well as differences when using different preparation methods (vacuum mixing versus mixing under atmospheric pressure). Staphylococci and Streptococci are most often found in periprosthetic joint infections [1]. The testing of Palacos R+G with 1.25% GS and CMW 1G with 2.5% GS in agar diffusion assays did not show significant differences for *Staphylococcus aureus* (*p* = 0.0546) and showed weak significance for *Staphylococcus epidermidis* (*p* = 0.0372) when applying a two-way ANOVA for repeated measures at different time points. In accordance with the literature [34], we saw a decrease in the release on day two (zone of inhibition testing), but the expected significant differences in the diameter of the inhibition areas of the two different cements were not clearly observable (Figure 1 and Figure 2) [5,10].

In order to study the dependency between GS concentration and area of inhibition diameters, 50 µL of GS standard (0.1 µg/mL–1.0 µg/mL) solutions was applied into holes in the agar plates [13]. The measured diameters showed a good correlation between concentrations and the diameters of the inhibition zones (R^2^ = 0.873, *p* < 0.0001, Figure 3). Once the results of our GS standard solution were reliable and showed a good correlation between GS concentration and the zone of inhibition diameter, eluates of two bone cements with different GS contents (2.5% and 1.25% GS) and the most remarkable differences in HPLC (CMW 1G and Palacos R+G, Figure 5) were chosen to run through the same setup. The application of the cement eluates showed more reliable results than the application of the cement discs on the surface of the agar plates (*p* < 0.0001). Combining our GS standard solution “calibration line” with the applied eluates of the two cements enabled us to calculate absolute amounts of discharged gentamicin at the chosen time points (Figure 4 and Figure 5) and enabled a comparison with HPLC.

The calculated release from the initial measurement (after 15 min) was around 200 µg in a total volume of 10 mL of buffer (around 40 µg/cm^2^) and a weight between 0.565 and 0.575 g.

Summing up the antibiotic release of the first three points of time in Figure 4 (0.25, 0.5 and 1.0), the calculated values for Palacos R+G are 75 µg/cm^2^ and 64 µg/cm^2^ for CMW 1G, which is in the range of the HPLC results (both around 50 µg/cm^2^). After 24 h and nine changes of the buffer (Figure 3), the released amount of GS from the 6th hour until the 24th hour was still 34 µg/cm^2^ for Palacos R+G and 18.4 µg/cm^2^ for CMW 1G.

HPLC as well as testing of eluates showed the best differences in the release kinetics. In our experiments, the 1.25% antibiotic-containing cement Palacos R+G showed better release over time than the 2.5% containing CMW 1G. These findings are also supported by Squire et al. (2011) and Kühn et al. (2016) and were also observed by other authors [9,13,28,33,34,35,36]. The differences may either stem from different compositions and ratios of the polymer and copolymer, the amount of incorporated active ingredient or the physical/chemical properties of the antibiotic [8,24,26,37]. Furthermore, the hydrophilic or hydrophobic character of the admixed antibiotic and their antibiotic combinations also have a considerable impact on elution properties [5,6,11,23].

## 5. Conclusions

In contrast to the method of direct application of cement discs onto agar, our data with eluates show remarkable differences in their inhibition areas. It is not recommended to test bone cement discs directly on the agar surface. Finally, in clinical situations, we should be aware of repeated purging during surgery, as it will wash away the locally high startup levels of the released antibiotics.

## Figures and Tables

**Table 1:** Testing overview for all materials and methods used for this study. SA (*Staphylococcus aureus*); SE (*Staphylococcus epidermidis*).

	CMW 1G	Palacos R+G	Gentamicin
Procedure			
	SA SE	SA SE	
Agar diffusion with discs	1–7 d	1–7 d	
Inhibition zone elution with eluates	0.25 h–14 d	0.25 h–14 d	
HPLC elution	1 h–7 d	1 h–7 d	
Agar diffusion-inhibition zone			0.0–1.0 µg

## Data Availability

All data are presented in this article. The data are also available through the Medical University of Graz (MUG).
